# Meat grinder injury of the arm: how to extract an arm safe, fast and pain free: case report and literature review

**DOI:** 10.1093/jscr/rjac001

**Published:** 2022-02-11

**Authors:** Ammar Al-Hassani, Bianca M Wahlen, Mohammed A M Ayasa, Suhail Hakim, Sherwan Khoschnau, Ayman El-Menyar, Hassan Al-Thani

**Affiliations:** Department of Surgery, Trauma Surgery, Hamad Medical Corporation, Doha, Qatar; Department of Anesthesiology, Hamad Medical Corporation, Doha, Qatar; Department of Anesthesiology, Hamad Medical Corporation, Doha, Qatar; Department of Surgery, Trauma Surgery, Hamad Medical Corporation, Doha, Qatar; Department of Surgery, Trauma Surgery, Hamad Medical Corporation, Doha, Qatar; Department of Surgery, Trauma Surgery, Clinical Research, Hamad Medical Corporation, Doha, Qatar; Clinical Medicine, Weill Cornell Medical College, Doha, Qatar; Department of Surgery, Trauma and Vascular Surgery, Hamad Medical Corporation, Doha, Qatar

## Abstract

Among the work-related injuries, meat grinder injuries are not uncommon. One of the major challenges in the trauma resuscitation room is the appropriate choice of anesthesia/analgesia when the patient is still conscious and the second challenge is to find the best way to early extract the patient’s limb out of the machine without adding more suffering. Herein, we presented a 23-year-old male patient who was brought fully conscious in a kneeling position with the right forearm entrapped in a big meat grinder machine with part of the crushed fingers being extruded out of the machine. The patient was in severe pain; however, his vital signs were stable. Analgo-sedation with Midazolam/Ketamine followed by ultrasound guided upper limb regional anesthesia was used and showed to be a fast and safe alternative in a conscious, not fasting patient when the extremity is still entrapped in a meat grinder machine.

## INTRODUCTION

In many countries, measures, such as ‘Occupational Safety and Health Act (OSH Act) or Injury and Illness prevention programs’, are in place. Consequently, these measures have caused a significant reduction in work-related injuries [1]. Nevertheless, work-related injuries still prevail [2]. However, the relevant authorities are continuously working on improving the preventive measures of work-related injuries [3].

Despite the continuous improvement of precautionary measures, these injuries still occur including meat grinder injuries. Many literatures have described meat grinder injuries as single case report or as case series [4, 5]. Such injuries can happen at any age group, but most described in the paediatric and early adolescent population [6]. Many case reports described that patients arriving to the trauma room conscious with the upper extremity still entrapped in the meat grinder [4, 5]. This mechanism of injury makes larger studies hard to conduct. Surprisingly, most of these patients immediately received general anaesthesia for the extraction of the upper extremity despite it is being commonly considered that trauma patients are not fasting and are therefore at a high risk for aspiration.

Furthermore, it is known from other areas of research that the use of Ketamine may influence, to some extent, phantom (stump) pain, while regional anesthesia may have a positive influence on such pain.

The underlying cause may be related to the patient position along with the fact that the patient’s extremity is still captured in a meat grinder. Typically, the patients are found in a flexed (bending forward) position with the arm stuck inside the grinder machine. To be able to apply a plexus anaesthesia, the patient and the grinder must be turned aside to allow a part of the upper limb plexus accessible for regional anesthesia. Normally, the possibility of an axillary plexus can be excluded to the fact that the machine with the captured arm cannot be positioned in such a way that the plexus can be reached safely. Interestingly, up to our knowledge, there are no descriptions of cases where the combination of analgo-sedation and plexus anaesthesia has been used to extract a trapped extremity out of a meat grinder machine.

## CASE PRESENTATION

**Figure 1:**
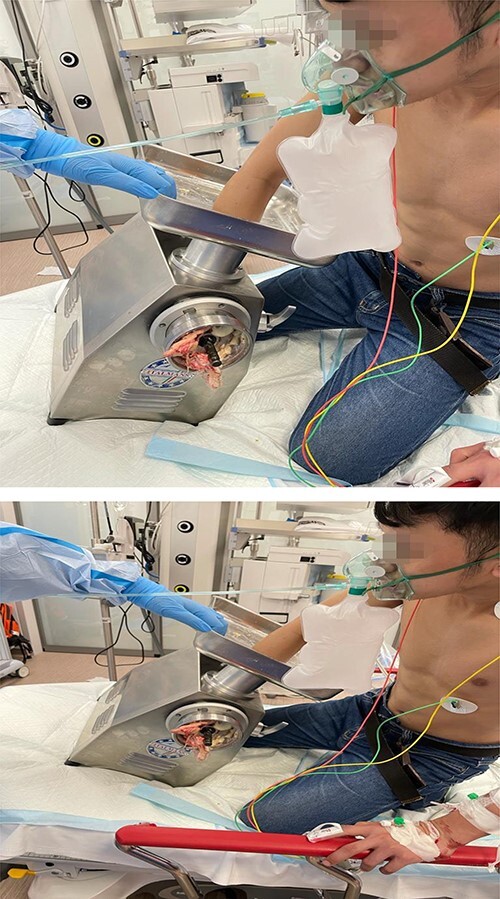
Shows the patient on arrival to the trauma room with the right upper limb trapped in the grinder machine.

**Figure 2:**
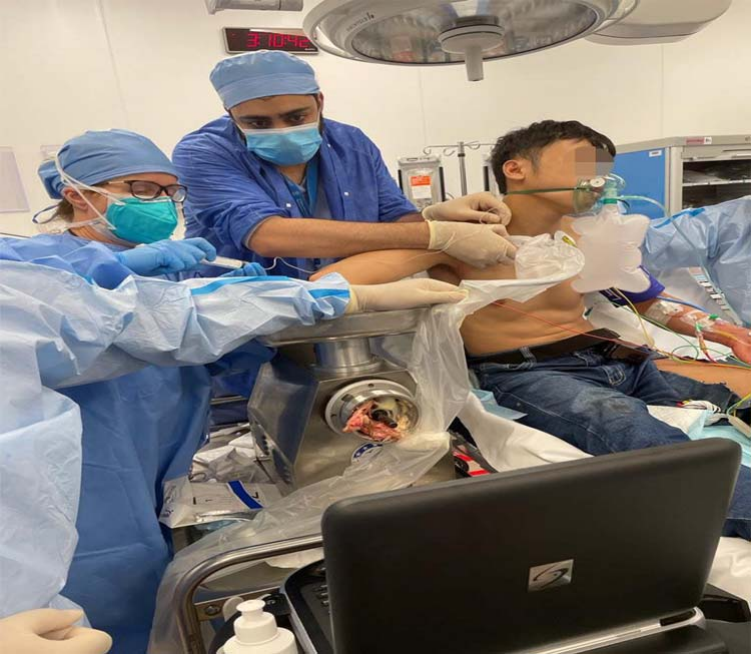
The patient is in a feasible position with ultrasound guided infraclavicular plexus block for extraction of the arm in place.

A 23-year-old male patient was brought to the trauma room by the emergency medical service. He was fully awake in agony, with a Glasgow Coma Scale 15/15. His right forearm was stuck in a meat grinder and parts of the hand were visible in the outlet of the machine. The patient was presented in a kneeling, bending forward, position, and the right arm at a 90° angle stuck in the machine. Figure 1 shows the patient on arrival to the trauma room with the right upper limb trapped in the grinder machine. To be able to reach the brachial plexus through the supra- or infraclavicular approach, the patient had to be turned by 180°. As the patient was not considered to be fasting, it had been decided to relieve his pain immediately. Therefore, a combination of Midazolam, Ketamine and regional anesthesia was chosen. Giving 1 mg of Midazolam in order to prevent the hallocinogenic effects of Ketamine, followed by 30 mg of S-Ketamine enabled the team to turn the patient by 180° to facilitate the access to the patient’s upper chest and to enable the anesthetist to give an ultrasound guided infraclavicular plexus block for extraction of the arm. Figure 2 shows that the patient is in a feasible position with ultrasound-guided infraclavicular plexus block for extraction of the arm in place.

The patient was put in a sitting position with the anesthetist standing behind him doing the ultrasound guided infraclavicular plexus block, as the supraclavicular part was not clearly visible, using a 15-ml lidocaine 2% and 5-ml levobupivacaine 0.5% under sterile conditions. After successful anesthesia, the arm was extracted by the civil defense. Vital signs were stable, and the patient was pain free. During reversal of the meat grinder machine, index-, middle- and ring fingers were found to have been already amputated. An X-ray of the right arm also revealed a distal fracture of the radius and ulna. After primary inspection and dressing from the plastic surgeon, the patient was transferred to the operating room, where he was intubated from the attending anesthetist despite the completely working plexus anesthesia, expecting a length of procedure which would extend beyond the duration of the given regional anesthesia. The patient underwent a debridement with k-wire fixation of all metacarpals and little finger proximal phalangeal fracture along with thenar muscle repair, stump closure of index, middle, ring finger and release of carpal tunnel with tagging suturing of the laceration. A splint was put in place and dressing was done again.

## DISCUSSION

 This is a rare case describing the use of regional anesthesia to early and safely extract an entrapped limb from a grinder machine. There are some hints in the literature that highlighted the favorable effect of Ketamine on the phantom pain, when given pre-or post-operatively [7]. In addition, studies showed evidence that, to a certain extent, stump or phantom limb pain can be alleviated by using the regional anesthesia technique [8–10].

Dertwinkel *et al.*[7] has shown that patients undergoing extremity amputation with regional anesthesia technique who were given ketamine intraoperatively and continued as infusion for 72 h post-operatively lead to a significant reduction of severe phantom limb pain through ketamine’s action on the dorsal horn nociceptive neurons. Lambert *et al.* [8] reported no statistically significant difference between epidural catheter and perineural catheter infusion of bupivacaine in the stump pain scores at long-term periods for patients undergoing general anesthesia for lower limb amputations, but Madabhushi and colleagues showed that patients, who were given perineural infusions of bupivacaine with clonidine for 96 h after lower limb amputations, had reported no stump pain or phantom limb pain for 12 months after surgery [9]. Recent studies have shown that ambulatory prolonged peripheral nerve block (up to 30 days post-amputation) may represent a new possible option to treat phantom pain and prevent the development of Phantom limb syndrome and chronic pain [10]. In 2017, Maajid *et al.* [11] described a patient with an arm entrapped in a meat mincer machine and in whom the supraclavicular block was used to salvage the limb. This report supports our approach in utilizing the regional anesthesia technique rather than general anesthesia in such situation. Therefore, the initial use of a catheter technique for plexus anesthesia as well as the administration of ketamine for long period would be beneficial.

## CONCLUSION

In trauma patients with entrapped limb in a machine, where an amputation or partial amputation is most likely to occur, the access for regional anesthesia for pain control is the key. This can be achieved with a catheter technique in combination with ketamine use as a bridge until the regional anesthesia works effectively. This is a safe and fast alternative to general anesthesia when performed in the emergency department with an expert team.

## ETHICS APPROVAL

This case report was granted approval from the medical research center, Hamad medical corporation, Doha, Qatar, with MRC-04-21-679.

## CONFLICT OF INTEREST STATEMENT

None declared.

## FUNDING

None.

## AUTHORS’ CONTRIBUTIONS

A.A., B.M.W., M.A., S.H., S.K. and H.A. all have substantial contribution in terms of patient care, data interpretation, writing and approval of this manuscript. A.E. contributed in interpretation, edited, revised and approved manuscript submission.
